# Supplementary material for: Evolution of the Electronic Structure of the trans-[Re6S8bipy4Cl2] Octahedral Rhenium Cluster during Reduction
Source: Molecules. 2023 Apr 23;28(9):3658. doi: 10.3390/molecules28093658 (PMC10180412; doi:10.3390/molecules28093658)
Supplement: Supplementary file 1 [file molecules-28-03658-s001.zip › molecules-2344849-supplementary.pdf]

Evolution of the electronic structure of the *trans*-[$Re_6S_8bipy_4Cl_2$] octahedral rhenium cluster during reduction

Maxim R. Ryzhikov*, Yakov M. Gayfulin, Anton A. Ulantikov,

Dmitry O. Arentov, Svetlana G. Kozlova, Yuri V. Mironov*

Nikolaev Institute of Inorganic Chemistry SB RAS, 3, Acad. Lavrentiev Ave., Novosibirsk, 630090, Russia

Table S1. The optimized coordinates of the [$Re_6S_8Cl_2bipy_4$]^n (n=0; S=0) cluster calculated at S12g/TZP/ZORA/DMSO level of theory.

Re	0.591012760	-1.384805510	1.055572150
Re	1.592484840	0.875890570	0.256764870
Re	-1.592484840	-0.875890570	-0.256764870
Re	-0.702868660	0.838898750	1.493838410
Re	0.702868660	-0.838898750	-1.493838410
S	2.676484320	-1.243240040	-0.148944430
S	-0.288682890	-2.873387380	-0.627668360
S	-1.576531350	-1.334167390	2.114477890
S	1.389015460	0.296403340	2.591347610
N	1.300441400	-3.051947090	2.317657070
Re	-0.591012760	1.384805510	-1.055572150
S	0.288682890	2.873387380	0.627668360
S	1.576531350	1.334167390	-2.114477890
N	3.513938920	1.919812870	0.564463720
S	-2.676484320	1.243240040	0.148944430
S	-1.389015460	-0.296403340	-2.591347610
N	-3.513938920	-1.919812870	-0.564463720
Cl	-1.629770540	1.945496070	3.461703610
Cl	1.629770540	-1.945496070	-3.461703610
C	2.527876630	-3.030401160	2.876086280
C	0.530465020	-4.136701160	2.541273730
N	-1.300441400	3.051947090	-2.317657070
C	4.557201680	1.717698840	-0.266164070
C	3.690289560	2.772223400	1.594558910
C	-4.557201680	-1.717698840	0.266164070
C	-3.690289560	-2.772223400	-1.594558910
H	3.132105480	-2.149623540	2.682275190
C	3.016142140	-4.067467780	3.649163590
C	0.955550670	-5.211522050	3.300381120
H	-0.457370080	-4.129275620	2.091168990
C	-2.527876630	3.030401160	-2.876086280
C	-0.530465020	4.136701160	-2.541273730
H	4.396540890	1.024820800	-1.086091070
C	5.778905960	2.342382530	-0.097615270
C	4.884873970	3.429495090	1.824816700
H	2.836106870	2.925808630	2.246791910

H	-4.396540890	-1.024820800	1.086091070
C	-5.778905960	-2.342382530	0.097615270
C	-4.884873970	-3.429495090	-1.824816700
H	-2.836106870	-2.925808630	-2.246791910
H	4.024656620	-3.987831270	4.046375060
C	2.230376870	-5.202915510	3.878384000
H	0.272975110	-6.044192180	3.448696900
H	-3.132105480	2.149623540	-2.682275190
C	-3.016142140	4.067467780	-3.649163590
C	-0.955550670	5.211522050	-3.300381120
H	0.457370080	4.129275620	-2.091168990
H	6.578888920	2.112382350	-0.796295970
C	5.974872660	3.225443400	0.970523360
H	4.945557740	4.115701910	2.665381240
H	-6.578888920	-2.112382350	0.796295970
C	-5.974872660	-3.225443400	-0.970523360
H	-4.945557740	-4.115701910	-2.665381240
C	2.727145330	-6.336715420	4.683660960
H	-4.024656620	3.987831270	-4.046375060
C	-2.230376870	5.202915510	-3.878384000
H	-0.272975110	6.044192180	-3.448696900
C	7.267745530	3.905534190	1.186072560
C	-7.267745530	-3.905534190	-1.186072560
C	3.597481620	-6.141870300	5.762402260
C	2.354956160	-7.655409150	4.397371350
C	-2.727145330	6.336715420	-4.683660960
C	8.094296110	4.263608140	0.114498030
C	7.723540500	4.218826050	2.471953720
C	-8.094296110	-4.263608140	-0.114498030
C	-7.723540500	-4.218826050	-2.471953720
H	3.913770590	-5.144031050	6.058899720
C	4.042365600	-7.243636860	6.484943460
C	2.860642820	-8.689240550	5.178036230
H	1.699355100	-7.887863090	3.560806530
C	-3.597481620	6.141870300	-5.762402260
C	-2.354956160	7.655409150	-4.397371350
H	7.798116400	4.073885260	-0.915031240
C	9.302674990	4.901971860	0.371172640
C	8.951592350	4.853698290	2.622318610
H	7.147627530	3.952176480	3.355628100
H	-7.798116400	-4.073885260	0.915031240
C	-9.302674990	-4.901971860	-0.371172640
C	-8.951592350	-4.853698290	-2.622318610
H	-7.147627530	-3.952176480	-3.355628100
N	3.694194720	-8.510867950	6.214113870
H	4.715597290	-7.098204180	7.331301400
H	2.581877020	-9.720340200	4.954390470
H	-3.913770590	5.144031050	-6.058899720
C	-4.042365600	7.243636860	-6.484943460
C	-2.860642820	8.689240550	-5.178036230
H	-1.699355100	7.887863090	-3.560806530
N	9.748324880	5.202203930	1.600591170
H	9.947853830	5.190618920	-0.460227420

H	9.316668430	5.092570730	3.622549590
N	-9.748324880	-5.202203930	-1.600591170
H	-9.947853830	-5.190618920	0.460227420
H	-9.316668430	-5.092570730	-3.622549590
N	-3.694194720	8.510867950	-6.214113870
H	-4.715597290	7.098204180	-7.331301400
H	-2.581877020	9.720340200	-4.954390470

Table S2. The optimized coordinates of the $[Re_6S_8Cl_2bipy_4]^n$ (n=2−; S=0) cluster calculated at S12g/TZP/ZORA/DMSO level of theory.

Re	0.603397000	-1.406137000	1.039026000
Re	1.592893000	0.885166000	0.271828000
Re	-1.592920000	-0.885149000	-0.271838000
Re	-0.708359000	0.808731000	1.505247000
Re	0.708341000	-0.808739000	-1.505299000
S	2.688710000	-1.221516000	-0.175424000
S	-0.266867000	-2.866310000	-0.676584000
S	-1.573441000	-1.374203000	2.095065000
S	1.384250000	0.263099000	2.599629000
N	1.307862000	-3.057257000	2.256315000
Re	-0.603354000	1.406098000	-1.039020000
S	0.266859000	2.866322000	0.676557000
S	1.573429000	1.374220000	-2.095089000
N	3.475215000	1.916648000	0.579740000
S	-2.688719000	1.221523000	0.175410000
S	-1.384251000	-0.263087000	-2.599653000
N	-3.475242000	-1.916620000	-0.579740000
Cl	-1.647946000	1.889043000	3.509480000
Cl	1.647921000	-1.889049000	-3.509523000
C	2.504566000	-3.014777000	2.901622000
C	0.578973000	-4.195858000	2.410444000
N	-1.307824000	3.057242000	-2.256332000
C	4.525031000	1.750093000	-0.270121000
C	3.676106000	2.755937000	1.630839000
C	-4.525060000	-1.750060000	0.270121000
C	-3.676138000	-2.755910000	-1.630840000
H	3.087150000	-2.108412000	2.766936000
C	2.982596000	-4.047161000	3.676049000
C	1.002415000	-5.267696000	3.162294000
H	-0.382608000	-4.217993000	1.906687000
C	-2.504525000	3.014759000	-2.901638000
C	-0.578935000	4.195838000	-2.410462000
H	4.363661000	1.074117000	-1.103987000
C	5.736534000	2.381606000	-0.105472000
C	4.863816000	3.412882000	1.857104000
H	2.832216000	2.891374000	2.300884000
H	-4.363689000	-1.074084000	1.103988000
C	-5.736563000	-2.381577000	0.105477000
C	-4.863849000	-3.412856000	-1.857101000
H	-2.832249000	-2.891351000	-2.300887000
H	3.960499000	-3.919716000	4.132945000
C	2.242773000	-5.240180000	3.838981000
H	0.336031000	-6.123195000	3.235314000

H	-3.087108000	2.108394000	-2.766953000
C	-2.982561000	4.047146000	-3.676059000
C	-1.002377000	5.267678000	-3.162313000
H	0.382646000	4.217974000	-1.906706000
H	6.515908000	2.166501000	-0.831862000
C	5.964457000	3.252120000	0.983438000
H	4.915295000	4.073732000	2.718377000
H	-6.515934000	-2.166472000	0.831870000
C	-5.964488000	-3.252095000	-0.983430000
H	-4.915328000	-4.073705000	-2.718374000
C	2.723370000	-6.359602000	4.642160000
H	-3.960466000	3.919703000	-4.132951000
C	-2.242737000	5.240163000	-3.838990000
H	-0.335993000	6.123177000	-3.235336000
C	7.239380000	3.930215000	1.190084000
C	-7.239409000	-3.930199000	-1.190069000
C	3.791810000	-6.233324000	5.555915000
C	2.152829000	-7.647966000	4.558131000
C	-2.723342000	6.359590000	-4.642164000
C	8.222275000	4.016687000	0.180143000
C	7.580247000	4.546369000	2.413706000
C	-8.222313000	-4.016647000	-0.180134000
C	-7.580262000	-4.546392000	-2.413676000
H	4.290816000	-5.280152000	5.715780000
C	4.211091000	-7.330204000	6.292611000
C	2.647813000	-8.680689000	5.339130000
H	1.337084000	-7.861882000	3.871332000
C	-3.791774000	6.233307000	-5.555925000
C	-2.152814000	7.647957000	-4.558116000
H	8.056512000	3.595004000	-0.808606000
C	9.419030000	4.670462000	0.426916000
C	8.807915000	5.173355000	2.559564000
H	6.905115000	4.524210000	3.266195000
H	-8.056565000	-3.594930000	0.808602000
C	-9.419061000	-4.670441000	-0.426896000
C	-8.807923000	-5.173395000	-2.559524000
H	-6.905125000	-4.524254000	-3.266162000
N	3.667937000	-8.560821000	6.211309000
H	5.035412000	-7.215973000	6.999655000
H	2.200585000	-9.673705000	5.258350000
H	-4.290768000	5.280131000	-5.715803000
C	-4.211063000	7.330191000	-6.292611000
C	-2.647807000	8.680684000	-5.339107000
H	-1.337077000	7.861873000	-3.871309000
N	9.746103000	5.256005000	1.595845000
H	10.166589000	4.734402000	-0.366671000
H	9.060206000	5.638641000	3.514827000
N	-9.746117000	-5.256029000	-1.595808000
H	-10.166625000	-4.734361000	0.366686000
H	-9.060201000	-5.638714000	-3.514775000
N	-3.667923000	8.560812000	-6.211292000
H	-5.035380000	7.215959000	-6.999661000
H	-2.200590000	9.673703000	-5.258314000

Table S3. The optimized coordinates of the $[Re_6S_8Cl_2bipy_4]^n$ (n=2−; S=1) cluster calculated at S12g/TZP/ZORA/DMSO level of theory.

Re	0.597408000	-1.397963000	1.051809000
Re	1.593469000	0.883664000	0.267772000
Re	-1.593469000	-0.883664000	-0.267772000
Re	-0.709666000	0.823562000	1.497068000
Re	0.709666000	-0.823562000	-1.497068000
S	2.684678000	-1.231673000	-0.155443000
S	-0.274350000	-2.870967000	-0.655720000
S	-1.578328000	-1.355174000	2.103769000
S	1.379663000	0.285494000	2.599733000
N	1.298487000	-3.042466000	2.281580000
Re	-0.597408000	1.397963000	-1.051809000
S	0.274350000	2.870967000	0.655720000
S	1.578328000	1.355174000	-2.103769000
N	3.476785000	1.916715000	0.582624000
S	-2.684678000	1.231673000	0.155443000
S	-1.379663000	-0.285494000	-2.599733000
N	-3.476785000	-1.916715000	-0.582624000
Cl	-1.653971000	1.920861000	3.488891000
Cl	1.653971000	-1.920861000	-3.488891000
C	2.525570000	-3.027247000	2.869396000
C	0.541752000	-4.154024000	2.491017000
N	-1.298487000	3.042466000	-2.281580000
C	4.533262000	1.746507000	-0.257488000
C	3.668393000	2.761324000	1.631474000
C	-4.533262000	-1.746507000	0.257488000
C	-3.668393000	-2.761324000	-1.631474000
H	3.126407000	-2.139433000	2.696498000
C	3.009533000	-4.063444000	3.634566000
C	0.968992000	-5.228485000	3.236679000
H	-0.442354000	-4.154317000	2.032117000
C	-2.525570000	3.027247000	-2.869396000
C	-0.541752000	4.154024000	-2.491017000
H	4.378419000	1.066230000	-1.089292000
C	5.743140000	2.378781000	-0.086680000
C	4.854638000	3.419019000	1.864425000
H	2.818997000	2.900641000	2.293544000
H	-4.378419000	-1.066230000	1.089292000
C	-5.743140000	-2.378781000	0.086680000
C	-4.854638000	-3.419019000	-1.864425000
H	-2.818997000	-2.900641000	-2.293544000
H	4.011208000	-3.960164000	4.043808000
C	2.243256000	-5.231872000	3.848834000
H	0.282493000	-6.062910000	3.354460000
H	-3.126407000	2.139433000	-2.696498000
C	-3.009533000	4.063444000	-3.634566000
C	-0.968992000	5.228485000	-3.236679000
H	0.442354000	4.154317000	-2.032117000
H	6.528771000	2.160596000	-0.805317000
C	5.961783000	3.254655000	1.000865000
H	4.899413000	4.084249000	2.722783000

H	-6.528771000	-2.160596000	0.805317000
C	-5.961783000	-3.254655000	-1.000865000
H	-4.899413000	-4.084249000	-2.722783000
C	2.732579000	-6.358412000	4.635538000
H	-4.011208000	3.960164000	-4.043808000
C	-2.243256000	5.231872000	-3.848834000
H	-0.282493000	6.062910000	-3.354460000
C	7.234400000	3.934972000	1.214688000
C	-7.234400000	-3.934972000	-1.214688000
C	3.832103000	-6.249052000	5.514062000
C	2.141330000	-7.638456000	4.567887000
C	-2.732579000	6.358412000	-4.635538000
C	8.220479000	4.026526000	0.208642000
C	7.568962000	4.547656000	2.441530000
C	-8.220479000	-4.026526000	-0.208642000
C	-7.568962000	-4.547656000	-2.441530000
H	4.348376000	-5.302595000	5.659059000
C	4.259664000	-7.353338000	6.234668000
C	2.648059000	-8.680058000	5.329154000
H	1.301113000	-7.838736000	3.906719000
C	-3.832103000	6.249052000	-5.514062000
C	-2.141330000	7.638456000	-4.567887000
H	8.058802000	3.607783000	-0.782068000
C	9.414757000	4.682600000	0.461880000
C	8.794601000	5.176988000	2.594036000
H	6.890556000	4.520422000	3.291321000
H	-8.058802000	-3.607783000	0.782068000
C	-9.414757000	-4.682600000	-0.461880000
C	-8.794601000	-5.176988000	-2.594036000
H	-6.890556000	-4.520422000	-3.291321000
N	3.697564000	-8.576393000	6.168025000
H	5.107438000	-7.252168000	6.915489000
H	2.185521000	-9.666939000	5.260176000
H	-4.348376000	5.302595000	-5.659059000
C	-4.259664000	7.353338000	-6.234668000
C	-2.648059000	8.680058000	-5.329154000
H	-1.301113000	7.838736000	-3.906719000
N	9.735892000	5.264960000	1.633805000
H	10.165024000	4.751230000	-0.328748000
H	9.042615000	5.639462000	3.551766000
N	-9.735892000	-5.264960000	-1.633805000
H	-10.165024000	-4.751230000	0.328748000
H	-9.042615000	-5.639462000	-3.551766000
N	-3.697564000	8.576393000	-6.168025000
H	-5.107438000	7.252168000	-6.915489000
H	-2.185521000	9.666939000	-5.260176000

Table S4. The optimized coordinates of the $[Re_6S_8Cl_2bipy_4]^n$ (n=4−; S=0) cluster calculated at S12g/TZP/ZORA/DMSO level of theory.

Re	0.581375000	-1.408903000	1.089525000
Re	1.593298000	0.886181000	0.323407000
Re	-1.614317000	-0.863138000	-0.229749000
Re	-0.714103000	0.824372000	1.550103000

Re	0.693559000	-0.801588000	-1.457137000
S	2.669943000	-1.233460000	-0.125186000
S	-0.300847000	-2.853250000	-0.641799000
S	-1.598778000	-1.351672000	2.141143000
S	1.370913000	0.264800000	2.651546000
N	1.259885000	-3.053717000	2.278507000
Re	-0.601336000	1.430412000	-0.998024000
S	0.278154000	2.876611000	0.731892000
S	1.580217000	1.372977000	-2.047602000
N	3.448147000	1.907825000	0.642516000
S	-2.690012000	1.257643000	0.216562000
S	-1.390932000	-0.244766000	-2.558339000
N	-3.471858000	-1.879758000	-0.549884000
Cl	-1.658587000	1.919282000	3.568646000
Cl	1.638328000	-1.896533000	-3.475560000
C	2.503038000	-3.087931000	2.854219000
C	0.485469000	-4.165306000	2.488364000
N	-1.276249000	3.063394000	-2.204613000
C	4.512921000	1.769654000	-0.208297000
C	3.642345000	2.766139000	1.692239000
C	-4.538253000	-1.734174000	0.297811000
C	-3.670837000	-2.732353000	-1.603326000
H	3.117268000	-2.205000000	2.700791000
C	2.978431000	-4.150398000	3.579315000
C	0.900355000	-5.259886000	3.201457000
H	-0.505364000	-4.139787000	2.044077000
C	-2.506218000	3.078741000	-2.808622000
C	-0.496853000	4.167999000	-2.432080000
H	4.368568000	1.083992000	-1.038185000
C	5.700325000	2.438696000	-0.058076000
C	4.804598000	3.463592000	1.901179000
H	2.804047000	2.874368000	2.374485000
H	-4.389026000	-1.055491000	1.132542000
C	-5.733373000	-2.387004000	0.138115000
C	-4.840646000	-3.414197000	-1.821401000
H	-2.830070000	-2.849068000	-2.281125000
H	3.983574000	-4.059465000	3.984125000
C	2.196792000	-5.324677000	3.793152000
H	0.194485000	-6.081574000	3.295423000
H	-3.128828000	2.206335000	-2.630947000
C	-2.957405000	4.109133000	-3.592971000
C	-0.887725000	5.230822000	-3.204396000
H	0.479216000	4.161788000	-1.955577000
H	6.470782000	2.245646000	-0.800633000
C	5.919800000	3.346657000	1.019726000
H	4.837125000	4.116610000	2.769992000
H	-6.504666000	-2.188915000	0.878488000
C	-5.959962000	-3.284691000	-0.946841000
H	-4.876509000	-4.064025000	-2.692470000
C	2.670914000	-6.466903000	4.528396000
H	-3.953040000	4.004764000	-4.017515000
C	-2.162624000	5.267260000	-3.843336000
H	-0.179153000	6.048832000	-3.309414000

C	7.148112000	4.075275000	1.199285000
C	-7.198753000	-3.991292000	-1.139947000
C	3.964928000	-6.531505000	5.121220000
C	1.891095000	-7.642529000	4.728245000
C	-2.603296000	6.365929000	-4.661076000
C	8.254155000	3.968401000	0.307813000
C	7.370112000	4.973917000	2.282142000
C	-8.309043000	-3.872274000	-0.255242000
C	-7.428887000	-4.877103000	-2.231729000
H	4.662656000	-5.700134000	5.045750000
C	4.373096000	-7.657940000	5.809763000
C	2.396732000	-8.716391000	5.435707000
H	0.882865000	-7.726372000	4.328123000
C	-3.873592000	6.398592000	-5.305466000
C	-1.809182000	7.523963000	-4.903131000
H	8.216542000	3.317931000	-0.563405000
C	9.410867000	4.692865000	0.524559000
C	8.568580000	5.650997000	2.405969000
H	6.605679000	5.149319000	3.036177000
H	-8.266121000	-3.229544000	0.621472000
C	-9.476799000	-4.574207000	-0.486120000
C	-8.638218000	-5.531696000	-2.369612000
H	-6.662387000	-5.059292000	-2.982079000
N	3.629100000	-8.774693000	5.994952000
H	5.372964000	-7.674964000	6.251254000
H	1.771933000	-9.603448000	5.569136000
H	-4.579383000	5.576858000	-5.204203000
C	-4.246139000	7.478985000	-6.082275000
C	-2.279321000	8.551629000	-5.698263000
H	-0.816795000	7.629051000	-4.469726000
N	9.617950000	5.545671000	1.556165000
H	10.240635000	4.588330000	-0.179238000
H	8.706324000	6.333533000	3.248538000
N	-9.691653000	-5.414714000	-1.526271000
H	-10.309646000	-4.460665000	0.212624000
H	-8.782130000	-6.204562000	-3.218910000
N	-3.487486000	8.577775000	-6.310092000
H	-5.227602000	7.471511000	-6.563463000
H	-1.644195000	9.425891000	-5.863284000

Table S5. The optimized coordinates of the $[Re_6S_8Cl_2bipy_4]^n$ (n=4−; S=1) cluster calculated at S12g/TZP/ZORA/DMSO level of theory.

Re	0.613519920	-1.410750910	1.034677760
Re	1.592957290	0.896945480	0.277739240
Re	-1.592957290	-0.896945480	-0.277739240
Re	-0.712010290	0.796134620	1.507073250
Re	0.712010290	-0.796134620	-1.507073250
S	2.696232600	-1.207721570	-0.181873340
S	-0.249987720	-2.866126200	-0.696402480
S	-1.564791400	-1.394817880	2.090944640
S	1.381621370	0.264932770	2.603775370
N	1.325446750	-3.045986580	2.233128200
Re	-0.613519920	1.410750910	-1.034677760

S	0.249987720	2.866126200	0.696402480
S	1.564791400	1.394817880	-2.090944640
N	3.442927370	1.924691800	0.598499220
S	-2.696232600	1.207721570	0.181873340
S	-1.381621370	-0.264932770	-2.603775370
N	-3.442927370	-1.924691800	-0.598499220
Cl	-1.669973040	1.870660530	3.536024260
Cl	1.669973040	-1.870660530	-3.536024260
C	2.563692500	-3.036509250	2.819987080
C	0.579779950	-4.174745990	2.451518040
N	-1.325446750	3.045986580	-2.233128200
C	4.516851700	1.771532810	-0.238404960
C	3.642612590	2.762933440	1.663465260
C	-4.516851700	-1.771532810	0.238404960
C	-3.642612590	-2.762933440	-1.663465260
H	3.157441220	-2.142131440	2.653104250
C	3.057723390	-4.068748280	3.574284470
C	1.013579560	-5.242757950	3.193326670
H	-0.404868160	-4.184459990	1.992381060
C	-2.563692500	3.036509250	-2.819987080
C	-0.579779950	4.174745990	-2.451518040
H	4.364104440	1.112350810	-1.087873750
C	5.724793920	2.391800310	-0.048764780
C	4.824840140	3.412627320	1.910607030
H	2.792891770	2.892464690	2.327683120
H	-4.364104440	-1.112350810	1.087873750
C	-5.724793920	-2.391800310	0.048764780
C	-4.824840140	-3.412627320	-1.910607030
H	-2.792891770	-2.892464690	-2.327683120
H	4.056081580	-3.943540940	3.986564440
C	2.302359990	-5.257860010	3.808909280
H	0.329830280	-6.082156940	3.294545560
H	-3.157441220	2.142131440	-2.653104250
C	-3.057723390	4.068748280	-3.574284470
C	-1.013579560	5.242757950	-3.193326670
H	0.404868160	4.184459990	-1.992381060
H	6.501730990	2.189733770	-0.782126590
C	5.957797740	3.260624330	1.057798910
H	4.861039640	4.051684190	2.789625220
H	-6.501730990	-2.189733770	0.782126590
C	-5.957797740	-3.260624330	-1.057798910
H	-4.861039640	-4.051684190	-2.789625220
C	2.793539530	-6.361806650	4.587264170
H	-4.056081580	3.943540940	-3.986564440
C	-2.302359990	5.257860010	-3.808909280
H	-0.329830280	6.082156940	-3.294545560
C	7.216138430	3.919375030	1.290974460
C	-7.216138430	-3.919375030	-1.290974460
C	4.074677770	-6.366797140	5.212425890
C	2.046162820	-7.555520600	4.806962890
C	-2.793539530	6.361806650	-4.587264170
C	8.344124500	3.766982340	0.434632890
C	7.450513310	4.782737560	2.399381700

C	-8.344124500	-3.766982340	-0.434632890
C	-7.450513310	-4.782737560	-2.399381700
H	4.748153580	-5.516745800	5.126030750
C	4.499167860	-7.454987820	5.949860160
C	2.566555420	-8.588307400	5.562903760
H	1.051604680	-7.683990280	4.384738740
C	-4.074677770	6.366797140	-5.212425890
C	-2.046162820	7.555520600	-4.806962890
H	8.300743930	3.134050270	-0.449150970
C	9.530818540	4.421695590	0.704006740
C	8.679448300	5.389937910	2.576270670
H	6.671865030	4.984739460	3.131954120
H	-8.300743930	-3.134050270	0.449150970
C	-9.530818540	-4.421695590	-0.704006740
C	-8.679448300	-5.389937910	-2.576270670
H	-6.671865030	-4.984739460	-3.131954120
N	3.785174270	-8.588280460	6.155507820
H	5.487198280	-7.425234900	6.416614540
H	1.966948990	-9.490382790	5.710697760
H	-4.748153580	5.516745800	-5.126030750
C	-4.499167860	7.454987820	-5.949860160
C	-2.566555420	8.588307400	-5.562903760
H	-1.051604680	7.683990280	-4.384738740
N	9.749244730	5.242478930	1.758907850
H	10.377397040	4.283279870	0.026562050
H	8.826468010	6.046882050	3.437403220
N	-9.749244730	-5.242478930	-1.758907850
H	-10.377397040	-4.283279870	-0.026562050
H	-8.826468010	-6.046882050	-3.437403220
N	-3.785174270	8.588280460	-6.155507820
H	-5.487198280	7.425234900	-6.416614540
H	-1.966948990	9.490382790	-5.710697760

Table S6. The optimized coordinates of the $[Re_6S_8Cl_2bipy_4]^n$ (n=4−; S=2) cluster calculated at S12g/TZP/ZORA/DMSO level of theory.

Re	0.613649000	-1.409796000	1.032300000
Re	1.586870000	0.895722000	0.279310000
Re	-1.586870000	-0.895722000	-0.279310000
Re	-0.714725000	0.794035000	1.508645000
Re	0.714725000	-0.794035000	-1.508645000
S	2.697899000	-1.203475000	-0.180318000
S	-0.246950000	-2.865290000	-0.699582000
S	-1.564744000	-1.399922000	2.087046000
S	1.379927000	0.263195000	2.604445000
N	1.327424000	-3.048100000	2.230573000
Re	-0.613649000	1.409796000	-1.032300000
S	0.246950000	2.865290000	0.699582000
S	1.564744000	1.399922000	-2.087046000
N	3.446144000	1.931693000	0.604708000
S	-2.697899000	1.203475000	0.180318000
S	-1.379927000	-0.263195000	-2.604445000
N	-3.446144000	-1.931693000	-0.604708000
Cl	-1.674568000	1.863395000	3.535624000

Cl	1.674568000	-1.863395000	-3.535624000
C	2.565221000	-3.037993000	2.818384000
C	0.582087000	-4.177365000	2.447609000
N	-1.327424000	3.048100000	-2.230573000
C	4.520589000	1.779301000	-0.230739000
C	3.643807000	2.767184000	1.671466000
C	-4.520589000	-1.779301000	0.230739000
C	-3.643807000	-2.767184000	-1.671466000
H	3.158893000	-2.143346000	2.652615000
C	3.059060000	-4.070320000	3.572611000
C	1.015908000	-5.245327000	3.189368000
H	-0.402165000	-4.187874000	1.987634000
C	-2.565221000	3.037993000	-2.818384000
C	-0.582087000	4.177365000	-2.447609000
H	4.368795000	1.121344000	-1.081552000
C	5.728235000	2.398596000	-0.039466000
C	4.824739000	3.416876000	1.922207000
H	2.792887000	2.895218000	2.334745000
H	-4.368795000	-1.121344000	1.081552000
C	-5.728235000	-2.398596000	0.039466000
C	-4.824739000	-3.416876000	-1.922207000
H	-2.792887000	-2.895218000	-2.334745000
H	4.056989000	-3.944792000	3.985782000
C	2.304035000	-5.259752000	3.806078000
H	0.332627000	-6.085206000	3.289523000
H	-3.158893000	2.143346000	-2.652615000
C	-3.059060000	4.070320000	-3.572611000
C	-1.015908000	5.245327000	-3.189368000
H	0.402165000	4.187874000	-1.987634000
H	6.505989000	2.197644000	-0.772307000
C	5.961007000	3.267147000	1.069982000
H	4.859331000	4.054391000	2.802451000
H	-6.505989000	-2.197644000	0.772307000
C	-5.961007000	-3.267147000	-1.069982000
H	-4.859331000	-4.054391000	-2.802451000
C	2.795091000	-6.363842000	4.584695000
H	-4.056989000	3.944792000	-3.985782000
C	-2.304035000	5.259752000	-3.806078000
H	-0.332627000	6.085206000	-3.289523000
C	7.216915000	3.925020000	1.305295000
C	-7.216915000	-3.925020000	-1.305295000
C	4.075595000	-6.368299000	5.210748000
C	2.047919000	-7.557653000	4.803417000
C	-2.795091000	6.363842000	-4.584695000
C	8.347329000	3.775090000	0.449849000
C	7.450842000	4.787633000	2.415834000
C	-8.347329000	-3.775090000	-0.449849000
C	-7.450842000	-4.787633000	-2.415834000
H	4.748790000	-5.517977000	5.125023000
C	4.499899000	-7.456500000	5.948390000
C	2.568066000	-8.590444000	5.559627000
H	1.053755000	-7.686348000	4.380376000
C	-4.075595000	6.368299000	-5.210748000

C	-2.047919000	7.557653000	-4.803417000
H	8.305400000	3.143548000	-0.435111000
C	9.532842000	4.429833000	0.721463000
C	8.678793000	5.394932000	2.594760000
H	6.671367000	4.988213000	3.148036000
H	-8.305400000	-3.143548000	0.435111000
C	-9.532842000	-4.429833000	-0.721463000
C	-8.678793000	-5.394932000	-2.594760000
H	-6.671367000	-4.988213000	-3.148036000
N	3.786097000	-8.589922000	6.153137000
H	5.487508000	-7.426516000	6.415981000
H	1.968704000	-9.492763000	5.706802000
H	-4.748790000	5.517977000	-5.125023000
C	-4.499899000	7.456500000	-5.948390000
C	-2.568066000	8.590444000	-5.559627000
H	-1.053755000	7.686348000	-4.380376000
N	9.750541000	5.249722000	1.778234000
H	10.380231000	4.292892000	0.044596000
H	8.824497000	6.050682000	3.457136000
N	-9.750541000	-5.249722000	-1.778234000
H	-10.380231000	-4.292892000	-0.044596000
H	-8.824497000	-6.050682000	-3.457136000
N	-3.786097000	8.589922000	-6.153137000
H	-5.487508000	7.426516000	-6.415981000
H	-1.968704000	9.492763000	-5.706802000

Table S7. The optimized coordinates of the $[Re_6S_8Cl_2bipy_4]^n$ (n=6−; S=0) cluster calculated at S12g/TZP/ZORA/DMSO level of theory.

Re	0.586510650	-1.385442480	1.107989470
Re	1.589376360	0.891777010	0.267329680
Re	-1.603436860	-0.901091140	-0.259532270
Re	-0.733033270	0.856077210	1.468886710
Re	0.718391690	-0.865874600	-1.461196130
S	2.681894800	-1.241514910	-0.095342930
S	-0.265360640	-2.896954140	-0.582368310
S	-1.620079600	-1.299352710	2.127445290
S	1.346034240	0.363016810	2.614937660
N	1.236220990	-2.933345730	2.404774410
Re	-0.599770360	1.376368970	-1.100153790
S	0.251152550	2.886890440	0.592053640
S	1.606648970	1.288774090	-2.120029970
N	3.426765890	1.918143360	0.570155440
S	-2.696086230	1.232618710	0.101472980
S	-1.360573060	-0.370754500	-2.607290840
N	-3.442269150	-1.925771190	-0.562971170
Cl	-1.718522570	2.024428560	3.461120380
Cl	1.702433730	-2.034996780	-3.453635700
C	2.505133350	-2.962317670	2.948512920
C	0.488644020	-4.071575110	2.633951230
N	-1.239112880	2.934004410	-2.389545160
C	4.521189140	1.738783750	-0.246686550
C	3.623080680	2.797607260	1.611832480
C	-4.536378680	-1.743649910	0.253911760

C	-3.641020590	-2.804604130	-1.604818890
H	3.097618380	-2.064173190	2.793136130
C	3.019647530	-4.020516940	3.644261600
C	0.940497790	-5.162400230	3.322756350
H	-0.518515990	-4.055689980	2.225110660
C	-2.500043630	2.963539340	-2.951274540
C	-0.487884390	4.071899010	-2.608051150
H	4.378570560	1.049431530	-1.074519510
C	5.725091610	2.362424000	-0.065042190
C	4.801543620	3.448080030	1.855914110
H	2.763106230	2.957397260	2.256706430
H	-4.391723950	-1.055613180	1.082479570
C	-5.742276500	-2.363082900	0.071551000
C	-4.821624200	-3.451044770	-1.849561500
H	-2.781180700	-2.966972130	-2.249321240
H	4.031892270	-3.908817770	4.029054850
C	2.267951560	-5.226804480	3.878321440
H	0.244313560	-5.991073200	3.439869970
H	-3.096038570	2.066615510	-2.802061360
C	-3.003208130	4.020645320	-3.656924370
C	-0.929652610	5.162906050	-3.302819650
H	0.513427220	4.055712080	-2.185273500
H	6.508608620	2.134887390	-0.785447990
C	5.960697990	3.272780840	1.023139290
H	4.824457000	4.115387270	2.715439620
H	-6.525060880	-2.133630060	0.792128700
C	-5.980873380	-3.271581830	-1.017590530
H	-4.846403430	-4.118126470	-2.709223100
C	2.775109890	-6.347187830	4.583352540
H	-4.010212450	3.909278660	-4.055340680
C	-2.247544230	5.226543560	-3.880834740
H	-0.231502740	5.991326250	-3.409488940
C	7.201190800	3.927258870	1.249937700
C	-7.224039370	-3.920427120	-1.245586950
C	4.106915830	-6.416665920	5.124080410
C	2.017122590	-7.543718870	4.837762180
C	-2.742265360	6.346026690	-4.595766580
C	8.358864940	3.751173500	0.417168220
C	7.436829990	4.836034330	2.337893120
C	-8.381947910	-3.738536130	-0.414221810
C	-7.462849070	-4.828448340	-2.333541140
H	4.801720590	-5.586206070	5.007449530
C	4.549591470	-7.533463090	5.798744660
C	2.561591470	-8.604168000	5.528977790
H	0.989907390	-7.639761240	4.488862350
C	-4.058447080	6.409053890	-5.174451290
C	-1.985040040	7.548210040	-4.825016410
H	8.331412040	3.089909520	-0.447524220
C	9.539390760	4.410384860	0.684244520
C	8.662516530	5.442405330	2.511179040
H	6.650282000	5.067793230	3.054458350
H	-8.352573440	-3.076843750	0.450090870
C	-9.565163580	-4.392446550	-0.682241950

C	-8.691099070	-5.429349060	-2.507758110
H	-6.676599720	-5.064308390	-3.049087080
N	3.825240540	-8.663234060	6.036253010
H	5.572047780	-7.539480360	6.188924200
H	1.944599080	-9.492025170	5.698397560
H	-4.750237350	5.572949720	-5.082899140
C	-4.488702220	7.525301480	-5.857852360
C	-2.516788190	8.608014310	-5.526866540
H	-0.969096820	7.650213590	-4.446018400
N	9.755783830	5.270540870	1.717560280
H	10.394580980	4.244998620	0.022020280
H	8.795387010	6.126833460	3.354416250
N	-9.784444560	-5.252147390	-1.715375370
H	-10.420337260	-4.222685320	-0.021093540
H	-8.826233890	-6.113552170	-3.350822690
N	-3.765663050	8.660926500	-6.070144630
H	-5.499005070	7.525385260	-6.278637380
H	-1.901065800	9.500661190	-5.674764360

Table S8. The optimized coordinates of the $[Re_6S_8Cl_2bipy_4]^n$ (n=6−; S=1) cluster calculated at S12g/TZP/ZORA/DMSO level of theory.

Re	0.597676920	-1.398348580	1.083738420
Re	1.596109050	0.890954520	0.269151380
Re	-1.600455890	-0.896114670	-0.263808900
Re	-0.719100850	0.837491840	1.483484290
Re	0.714653100	-0.842884430	-1.478215750
S	2.689092450	-1.232766680	-0.131971530
S	-0.267573170	-2.886331720	-0.625302790
S	-1.595602030	-1.328751340	2.120114560
S	1.363980090	0.322886200	2.611115850
N	1.281598520	-2.990762140	2.310111600
Re	-0.601548690	1.393063860	-1.078482530
S	0.262970590	2.880840320	0.630985410
S	1.591814760	1.323199070	-2.114815480
N	3.434715010	1.913358750	0.574539840
S	-2.693362780	1.227942400	0.136470360
S	-1.368317940	-0.327915450	-2.605703470
N	-3.439989830	-1.917304030	-0.568868140
Cl	-1.691705640	1.979952310	3.494102520
Cl	1.686678740	-1.985674850	-3.488936830
C	2.536855610	-3.004102930	2.882367330
C	0.523600310	-4.116017620	2.561274620
N	-1.281891070	2.986562260	-2.304970750
C	4.527190550	1.740686930	-0.247670990
C	3.636021130	2.784216060	1.623397700
C	-4.532708760	-1.742047680	0.252557370
C	-3.642668400	-2.787552460	-1.617991740
H	3.136571060	-2.114162340	2.709587280
C	3.033995990	-4.042418370	3.619980700
C	0.957860970	-5.187020200	3.291919800
H	-0.474260240	-4.110266650	2.130214750
C	-2.531924500	2.997691220	-2.888417190
C	-0.524543710	4.114174840	-2.547342120

H	4.380837650	1.060624290	-1.082555540
C	5.732174710	2.360761490	-0.064310100
C	4.813611550	3.435999890	1.865866750
H	2.780191030	2.934305180	2.276253190
H	-4.385095450	-1.063150590	1.088160620
C	-5.739407440	-2.358272310	0.067553030
C	-4.821937830	-3.435818360	-1.861869430
H	-2.786528620	-2.940043800	-2.269905680
H	4.041464140	-3.924988230	4.015082960
C	2.274185330	-5.238871910	3.872772760
H	0.259103300	-6.012616550	3.414282400
H	-3.131276510	2.106351780	-2.721408420
C	-3.024503110	4.035454440	-3.629926050
C	-0.955163270	5.185599980	-3.279320960
H	0.469655400	4.109973990	-2.107999130
H	6.513496650	2.136866250	-0.788375300
C	5.971898440	3.266962920	1.028146890
H	4.838815080	4.097594960	2.729857050
H	-6.520689070	-2.132597600	0.791110590
C	-5.980882900	-3.262969300	-1.025772430
H	-4.848125760	-4.097273780	-2.725939940
C	2.770285550	-6.346603870	4.607655870
H	-4.028175720	3.916228520	-4.034072110
C	-2.265624440	5.234494310	-3.873384390
H	-0.257291680	6.013023620	-3.393931790
C	7.210816100	3.920930700	1.253401110
C	-7.221904370	-3.912238660	-1.253061010
C	4.094441560	-6.406729320	5.165605710
C	2.008006290	-7.537190990	4.871573160
C	-2.756974290	6.342258520	-4.611328820
C	8.366650380	3.752840400	0.414274760
C	7.451438350	4.824220250	2.346499550
C	-8.378680750	-3.739217300	-0.416197960
C	-7.464071610	-4.815000110	-2.346262570
H	4.790218960	-5.577659940	5.045786250
C	4.529253950	-7.513756670	5.861420550
C	2.544117430	-8.587908170	5.584233330
H	0.984493510	-7.637307910	4.513253990
C	-4.071120110	6.395728650	-5.193047040
C	-1.998743500	7.539631530	-4.855616150
H	8.336878530	3.096908790	-0.454518390
C	9.546744780	4.412516830	0.679995030
C	8.676476030	5.431697980	2.517393720
H	6.668081120	5.050385810	3.068486010
H	-8.348113460	-3.082748480	0.452159520
C	-9.560763740	-4.394582090	-0.683687320
C	-8.691068670	-5.417998150	-2.518999080
H	-6.680299560	-5.044472180	-3.066749940
N	3.801650370	-8.639314230	6.105997490
H	5.546943400	-7.514412510	6.263715590
H	1.925030280	-9.472946360	5.759986830
H	-4.762326100	5.560394920	-5.091726640
C	-4.501344430	7.503291820	-5.890830550

C	-2.529880100	8.590571400	-5.571543520
H	-0.982854790	7.645659310	-4.477805360
N	9.767362490	5.267500860	1.717693080
H	10.399103000	4.252460350	0.012654090
H	8.811654940	6.111466570	3.364155200
N	-9.782744680	-5.249318140	-1.721304470
H	-10.413749310	-4.230839350	-0.018037880
H	-8.827339030	-6.097608090	-3.365714530
N	-3.778077110	8.635723570	-6.115748640
H	-5.511181100	7.498273730	-6.312438940
H	-1.914390040	9.481288270	-5.730712800

Table S9. The optimized coordinates of the $[Re_6S_8Cl_2bipy_4]^n$ (n=8−; S=0) cluster calculated at S12g/TZP/ZORA/DMSO level of theory.

Re	0.591394510	-1.389331520	1.105634650
Re	1.593559490	0.893449360	0.264962170
Re	-1.604207580	-0.901375490	-0.259278010
Re	-0.727997410	0.853728430	1.470608850
Re	0.716852940	-0.862136340	-1.464983850
S	2.687721390	-1.241041980	-0.107383090
S	-0.265331800	-2.899247120	-0.592382610
S	-1.616234410	-1.301605860	2.130940500
S	1.352107830	0.361307770	2.615296940
N	1.248929810	-2.937137380	2.372192290
Re	-0.601419930	1.381417960	-1.099964030
S	0.254552600	2.890592820	0.599761350
S	1.606166350	1.292347770	-2.125596490
N	3.414780260	1.911256630	0.563199680
S	-2.698465950	1.233328240	0.111468030
S	-1.363211160	-0.367707950	-2.609492880
N	-3.426547050	-1.917651690	-0.557999690
Cl	-1.723982070	2.038131860	3.496952170
Cl	1.711379100	-2.047309520	-3.491396010
C	2.516627660	-2.965553080	2.935720270
C	0.496636300	-4.076717820	2.621444140
N	-1.252776350	2.934870650	-2.362347830
C	4.520279170	1.736243420	-0.253263710
C	3.620614480	2.797146280	1.608208110
C	-4.532016990	-1.739897350	0.257974090
C	-3.634260530	-2.802631360	-1.603367160
H	3.111397820	-2.068994250	2.775702390
C	3.025831700	-4.014389540	3.644560430
C	0.941425660	-5.160158280	3.321312720
H	-0.509958440	-4.062558040	2.209653430
C	-2.513230810	2.963856050	-2.941249910
C	-0.497766380	4.075125630	-2.600501500
H	4.376672900	1.051849910	-1.085888110
C	5.724260930	2.352396650	-0.069771240
C	4.794887830	3.448460010	1.852093080
H	2.760432020	2.959100660	2.253354060
H	-4.386859540	-1.056648410	1.091262770
C	-5.737754010	-2.352186800	0.073360820
C	-4.810342590	-3.450447300	-1.848241570

H	-2.773970150	-2.967033700	-2.247819150
H	4.036477090	-3.895753230	4.034371820
C	2.276152770	-5.233899670	3.888645520
H	0.239049880	-5.984140590	3.444144150
H	-3.110659270	2.067868940	-2.787586700
C	-3.013183070	4.012612540	-3.656905230
C	-0.933948510	5.158964830	-3.304893400
H	0.503387410	4.061108000	-2.175888210
H	6.505599260	2.120494500	-0.793046860
C	5.975738150	3.274404600	1.024159880
H	4.810791620	4.120094320	2.709940580
H	-6.518668280	-2.118667760	0.796561110
C	-5.991505790	-3.272577470	-1.021421350
H	-4.827620460	-4.122018430	-2.706124640
C	2.776330010	-6.340257110	4.588267710
H	-4.019242610	3.894397700	-4.058533820
C	-2.260400350	5.232239140	-3.891032720
H	-0.230499300	5.983548080	-3.416876820
C	7.199622360	3.916339880	1.250995180
C	-7.217535530	-3.910040360	-1.249182150
C	4.112228110	-6.417219720	5.151187640
C	2.025696250	-7.556578340	4.841986360
C	-2.750620770	6.339182880	-4.596776180
C	8.380405210	3.746246260	0.422264770
C	7.452531040	4.836440870	2.346151180
C	-8.398126480	-3.735461660	-0.421011180
C	-7.473305310	-4.829609500	-2.344158700
H	4.807448430	-5.584196320	5.046906960
C	4.550734830	-7.532227450	5.825501250
C	2.570193230	-8.612715170	5.533915600
H	1.001231450	-7.661536710	4.484553070
C	-4.075883800	6.414726360	-5.184353200
C	-1.998090910	7.557706390	-4.833694510
H	8.360157900	3.085332300	-0.444419900
C	9.556902730	4.403027580	0.694159370
C	8.676802740	5.437168810	2.519629600
H	6.667932040	5.073566680	3.064883480
H	-8.375955650	-3.074303670	0.445425730
C	-9.576925530	-4.387806850	-0.693417460
C	-8.699680620	-5.425982630	-2.517979620
H	-6.689116080	-5.070121830	-3.062228290
N	3.834041710	-8.676295290	6.059285470
H	5.570919550	-7.530526350	6.224948360
H	1.952708230	-9.503599230	5.693248490
H	-4.770746400	5.579510610	-5.096415940
C	-4.504299530	7.530674810	-5.863543140
C	-2.532310800	8.614821570	-5.531975710
H	-0.980684520	7.664232150	-4.457111840
N	9.785378450	5.270137690	1.730944160
H	10.412201510	4.232830440	0.030886020
H	8.804631470	6.121380960	3.365686850
N	-9.808127270	-5.254423660	-1.730026980
H	-10.431971250	-4.213943800	-0.030783070

H	-8.829557340	-6.110186910	-3.363759650
N	-3.786259010	8.677136420	-6.080541100
H	-5.516553420	7.527427630	-6.282702960
H	-1.913995150	9.507610450	-5.676995060

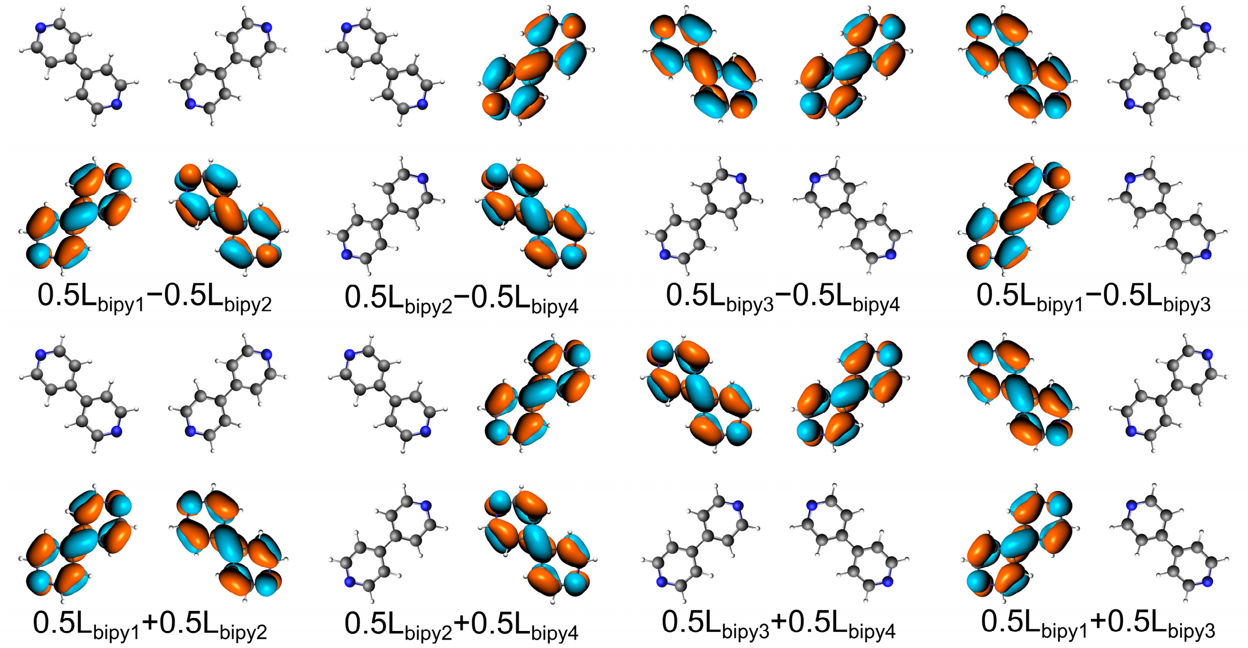

Figure S1. The bonding (bottom line) and antibonding (top line) hypotetical MOs in $bipy_4$ fragment composed from LUMO of bipy ligands in cis-positions.

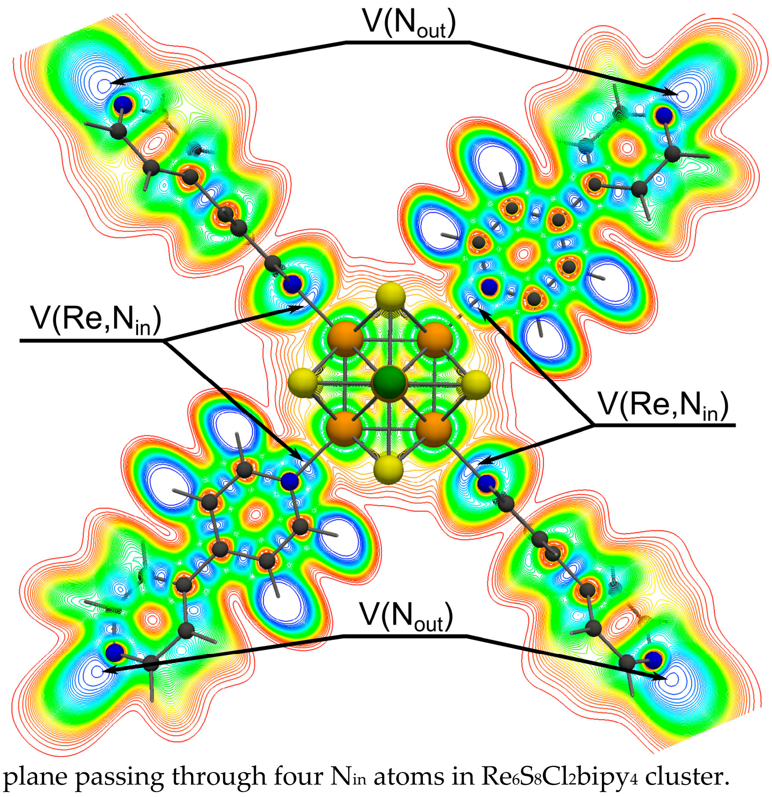

Figure S2. ELF slice plane passing through four N_{in} atoms in $Re_6S_8Cl_2bipy_4$ cluster.

Table S10. The average QTAIM charges for $[Re_6S_8Cl_2bipy_4]^n$ (n=0, 2–, 4–, 6–, 8–) cluster.

**n**	**0**	**2–**	**4–**	**6–**	**8–**
**S**	**0**	**1**	**2**	**1**	**0**
Re	1.08	1.08	1.08	1.08	1.09
S	-0.71	-0.74	-0.75	-0.80	-0.81
Cl	-0.71	-0.74	-0.75	-0.78	-0.80
N	-1.16	-1.15	-1.17	-1.16	-1.18
N_{term}	-1.10	-1.10	-1.13	-1.15	-1.18
C	0.19	0.16	0.13	0.10	0.08
H	0.07	0.05	0.03	0.02	-0.01
bipy	0.17	-0.26	-0.75	-1.15	-1.63
